# Evaluating in vivo effectiveness of sotrovimab for the treatment of Omicron subvariant BA.2 versus BA.1: a multicentre, retrospective cohort study

**DOI:** 10.1186/s13104-024-06695-x

**Published:** 2024-01-24

**Authors:** Carson K. L. Lo, Calvin K. F. Lo, Adam S. Komorowski, Victor Leung, Nancy Matic, Susan McKenna, Santiago Perez-Patrigeon, Prameet M. Sheth, Christopher F. Lowe, Zain Chagla, Anthony D. Bai

**Affiliations:** 1 Division of Infectious Diseases, Department of Medicine, McMaster University, Hamilton, ON Canada; 2 Transplant Infectious Diseases and Ajmera Transplant Centre, University Health Network, 585 University Avenue, MaRS Building, 9th Floor, Toronto, ON M5G 2N2 Canada; 3 Department of Pathology and Laboratory Medicine, University of British Columbia, Vancouver, BC Canada; 4 Department of Health Research Methods, Evidence, and Impact, McMaster University, Hamilton, ON Canada; 5 Medical Microbiology, Department of Pathology and Molecular Medicine, McMaster University, Hamilton, ON Canada; 6 Division of Medical Microbiology and Virology, St. Paul’s Hospital, Providence Health Care, Vancouver, BC Canada; 7 Department of Pharmacy Services, Kingston Health Sciences Centre, Kingston, ON Canada; 8 Division of Infectious Diseases, Department of Medicine, Queen’s University, Kingston, ON Canada; 9 Division of Microbiology, Kingston Health Sciences Centre, Kingston, ON Canada; 10 Department of Pathology and Molecular Medicine, Queen’s University, Kingston, ON Canada; 11 Department of Biomedical & Molecular Sciences, Queen’s University, Kingston, ON Canada; 12 Gastrointestinal Disease Research Unit, Kingston Health Sciences Centre, Kingston, ON Canada; 13 St. Joseph’s Healthcare Hamilton, Hamilton, ON Canada

**Keywords:** Sotrovimab, SARS-CoV-2, COVID-19, COVID-19 drug treatment, Omicron, BA.2 subvariant, Antibodies, Monoclonal

## Abstract

**Background:**

In vitro data suggested reduced neutralizing capacity of sotrovimab, a monoclonal antibody, against Omicron BA.2 subvariant. However, limited in vivo data exist regarding clinical effectiveness of sotrovimab for coronavirus disease 2019 (COVID-19) due to Omicron BA.2.

**Methods:**

A multicentre, retrospective cohort study was conducted at three Canadian academic tertiary centres. Electronic medical records were reviewed for patients ≥ 18 years with mild COVID-19 (sequencing-confirmed Omicron BA.1 or BA.2) treated with sotrovimab between February 1 to April 1, 2022. Thirty-day co-primary outcomes included hospitalization due to moderate or severe COVID-19; all-cause intensive care unit (ICU) admission, and all-cause mortality. Risk differences (BA.2 minus BA.1 group) for co-primary outcomes were adjusted with propensity score matching (e.g., age, sex, vaccination, immunocompromised status).

**Results:**

Eighty-five patients were included (15 BA.2, 70 BA.1) with similar baseline characteristics between groups. Adjusted risk differences were non-statistically significant between groups for 30-day hospitalization (− 14.3%; 95% confidence interval (CI): − 32.6 to 4.0%), ICU admission (− 7.1%; 95%CI: − 20.6 to 6.3%), and mortality (− 7.1%; 95%CI: − 20.6 to 6.3%).

**Conclusions:**

No differences were demonstrated in hospitalization, ICU admission, or mortality rates within 30 days between sotrovimab-treated patients with BA.1 versus BA.2 infection. More real-world data may be helpful to properly assess sotrovimab’s effectiveness against infections due to specific emerging COVID-19 variants.

**Supplementary Information:**

The online version contains supplementary material available at 10.1186/s13104-024-06695-x.

## Introduction

Coronavirus disease 2019 (COVID-19) treatment have rapidly changed over time [1]. In particular, many monoclonal antibody treatments for COVID-19 have come and go [2], quickly withdrawn from recommendations as new strains showed reduced neutralizing activity based on in vitro studies [3].

As an example, sotrovimab, a monoclonal antibody, was once a favoured therapeutic option for COVID-19 given its therapeutic advantages versus other approved drug options, requiring only a single intravenous dose with fewer drug-drug interactions [4]. An in vitro study demonstrated reduced neutralizing capacity of sotrovimab against Omicron BA.2, where the FNRT_50_ value (titer of sotrovimab required for 50% reduction in number of infectious foci) was approximately 50-fold higher for BA.2 compared to the SARS-CoV-2 ancestral strain [3]. By March 2022, BA.2 represented about 25% of confirmed COVID-19 cases in Ontario, Canada [5]. In response, both United States and Canadian (Ontario) guidelines revoked recommendations on therapeutic use of sotrovimab for COVID-19 by early April 2022 [6, 7]. However, the question remains whether in vitro data translated to decreased efficacy in vivo of sotrovimab against BA.2.

As BA.2 emerged before large-scale discontinuation of sotrovimab in clinical practice, a few observational studies had been able to evaluate BA.2-infected patients who received sotrovimab as treatment [8–10]. These studies demonstrated no significant differences in hospitalizations in BA.1 versus BA.2-infected patients treated with sotrovimab, although the analyses were either unadjusted for baseline risks or adjusted for age and immunization status only. In Canada, mild COVID-19 patients who met criteria to receive sotrovimab were considered high risk either from immunocompromising conditions or having risk factors for progression to severe disease, per local (e.g., Ontario, British Columbia) guidelines (Additional file 1: Text S1) [4]. Adjusting for potential bias from age, vaccination status, as well as immunocompromised status and risk factors for severe COVID-19 may provide a better estimate of effectiveness of sotrovimab between variants in infected individuals.

To our knowledge, there is no existing data that used a propensity-matched analysis to compare the effectiveness of sotrovimab in patients with mild COVID-19 who were infected with Omicron BA.2 versus those infected with Omicron BA.1. We conducted a retrospective cohort study in patients with mild COVID-19 due to Omicron collecting local Canadian patient data. Our primary objective was to compare the effectiveness of sotrovimab in confirmed BA.2 cases versus BA.1 cases in terms of hospitalization, intensive care unit (ICU) admission and mortality risk.

## Methods

### Study design and participants

We conducted a multicentre retrospective cohort study in three Canadian academic tertiary care centres. Patients aged ≥ 18 years who received single-dose intravenous sotrovimab (500 mg) as treatment for mild COVID-19 were included. Based on the treatment algorithms from local guidelines [4], patients would have only received sotrovimab as COVID-19 directed therapy, and thus would not have received any other therapeutics (e.g., remdesivir, nirmatrelvir/ritonavir). The study period February 1 to April 1, 2022 was chosen to capture then-predominant BA.1 and BA.2 cases who received sotrovimab as treatment prior to its discontinuation within Canada. Mild COVID-19 was defined as those not requiring additional supplementary oxygen from their clinical baseline as per local and international guidelines [4, 11]. Initial diagnosis was established by rapid antigen test or real-time reverse transcriptase-polymerase chain reaction (PCR) from upper or lower respiratory tract specimens. Omicron BA.1 or BA.2 confirmation via whole genome sequencing (WGS) or targeted single-nucleotide polymorphism (SNP) PCR was required for inclusion.

### Data collection

Patient data were collected retrospectively from medical records for baseline demographic characteristics, prior COVID-19 infection and vaccination status, symptoms before receiving sotrovimab, immunocompromised status, and comorbidities at high risk for complication or severe COVID-19, as derived from local guidelines (Additional file 1: Text S1) [4]. Omicron subvariant WGS or targeted SNP PCR results were provided by the microbiology laboratory of each respective study centre.

Co-primary outcomes within 30 days of sotrovimab administration included (1) hospitalization due to moderate or severe COVID-19; (2) all-cause admission to ICU, and; (3) all-cause mortality. Investigators were blinded to subvariant status during the data entry process.

### Statistical analysis

For descriptive analysis, continuous (mean and standard deviation) and categorical variables (counts and percentages) were used. BA.1 and BA.2 groups were compared using Student’s t-test for continuous variables and Fisher’s exact test for categorical variables. For co-primary outcomes, risk difference of BA.2 minus BA.1 group was calculated with estimated two-sided 95% confidence intervals (CI) based on the method of Agresti and Caffo [12].

To address potential bias, propensity score for BA.2 subvariant was estimated by logistic regression of the following prognostic factors determined a priori: age, sex, vaccination status, immunocompromised status, and number of risk factors for progression to severe disease. BA.2 and BA.1 cases were matched using nearest neighbour matching with a specified caliper width of 0.3 times the standard deviation of the logit of propensity scores. Standardized mean difference was used to assess for balance of prognostic factors. Risk difference and two-sided 95% CI were estimated for each outcome; CI estimates were calculated for matched patients using the method of Agresti and Min [13, 14].

All tests were two-sided, with significance defined as P < 0.05. All analyses were done using statistical software R (version 4.1.2), with statistical packages DescTools and MatchIt for risk difference CI and propensity score matching, respectively [15, 16].

## Results

Eighty-five patients with COVID-19 (70 BA.1, 15 BA.2) were included with similar baseline characteristics (Table 1). None of the patients have received any other COVID-19 therapeutics or adjunctive therapies prior to or at the time of receiving sotrovimab. Co-primary outcomes within 30 days of sotrovimab administration are presented in Table 2 and Additional file 1: Fig. S1. Following matching by propensity scores (14 BA.1 and 14 BA.2 patients), the maximum standardized difference was 0.0839, suggesting good balance of baseline characteristics (Additional file 1: Table S1). The adjusted risk difference at 30 days for BA.2 group minus BA.1 group was (Table 3 and Additional file 1: Fig. S2): − 14.3% (95% CI: − 32.6 to 4.0%) for hospitalization; − 7.1% (95% CI: − 20.6 to 6.3%) for ICU admission; and − 7.1% (95% CI: − 20.6 to 6.3%) for death.

**Table 1:** Baseline patient descriptives and clinical risk factors for severe COVID-19 infection

	BA.1 (n = 70)	BA.2 (n = 15)	P value
Age, mean in years (standard deviation, SD)	58.5 (19.8)	61.0 (16.2)	0.613
Female	37 (52.9%)	4 (26.7%)	0.089
Received 2 or more doses of COVID-19 vaccines	56 (80.0%)	13 (86.7%)	0.726
Prior COVID-19 infection	0 (0%)	0 (0%)	N/A
Immunocompromised	47 (67.1%)	9 (60.0%)	0.765
Solid organ transplant	20 (28.6%)	6 (40.0%)	0.376
Haematopoietic stem cell transplant	2 (2.9%)	0 (0%)	> 0.999
Haematologic malignancy	7 (10.0%)	0 (0%)	0.344
Solid tumour cancer	6 (8.6%)	0 (0%)	0.585
Primary immunodeficiency	1 (1.4%)	0 (0%)	> 0.999
HIV/AIDS	0 (0%)	0 (0%)	N/A
High dose steroids (≥ 20 mg prednisone equivalent > 2 weeks)	10 (14.3%)	3 (20.0%)	0.693
Chemotherapy	8 (11.4%)	0 (0%)	0.340
Biologic for rheumatic diseases	8 (11.4%)	1 (6.7%)	> 0.999
Conventional DMARD for rheumatic diseases	19 (27.1%)	6 (40.0%)	0.357
Number of risk factors for progression to severe disease, mean (SD)	1.9 (1.7)	3.1 (2.0)	0.052
Obesity	13 (18.6%)	1 (6.7%)	0.447
Diabetes mellitus	21 (30.0%)	6 (40.0%)	0.544
Hypertension	31 (44.3%)	10 (66.7%)	0.157
Coronary artery disease	11 (15.7%)	7 (46.7%)	0.014
Congestive heart failure	5 (7.1%)	6 (40.0%)	0.003
Chronic respiratory disease	17 (24.3%)	3 (20.0%)	> 0.999
Cerebral palsy	0 (0%)	0 (0%)	N/A
Intellectual disability	5 (7.1%)	1 (6.7%)	> 0.999
Sickle cell disease	0 (0%)	0 (0%)	N/A
Chronic kidney disease, eGFR < 60 mL/min	24 (34.3%)	8 (53.3%)	0.240
Liver disease	6 (8.6%)	4 (26.7%)	0.070
Pregnancy	2 (2.9%)	0 (0%)	> 0.999
Symptoms, days before sotrovimab. Mean (SD)	3.9 (1.6)	3.6 (1.5)	0.416

**Table 2:** Risk differences of co-primary outcomes 30 days post-sotrovimab (BA.1 vs. BA.2)

Outcomes within 30 days	BA.1 (n = 70)	BA.2 (n = 15)	Risk difference, BA.2 minus BA.1 (95% CI)	P value^a^
Hospitalization due to COVID-19	8 (11.4%)	1 (6.7%)	− 4.7% (− 17.9 to 16.4%)	> 0.9999
All-cause ICU admission	3 (4.3%)	0 (0%)	− 4.3% (− 12.0 to 12.7%)	> 0.9999
All-cause mortality	4 (5.7%)	0 (0%)	− 5.7% (− 13.7 to 11.6%)	> 0.9999

^a^p values computed from Fisher’s exact test

**Table 3:** Adjusted risk differences for co-primary outcomes after matching by propensity score^a^

Outcomes within 30 days	BA.1 (n = 14)	BA.2 (n = 14)	Adjusted risk difference, BA.2 minus BA.1 (95% CI)	P value^b^
Hospitalization due to COVID-19	3 (21.4%)	1 (7.1%)	− 14.3% (− 32.6 to 4.0%)	0.5956
All-cause ICU admission	1 (7.1%)	0 (0%)	− 7.1% (− 20.6 to 6.3%)	> 0.9999
All-cause mortality	1 (7.1%)	0 (0%)	− 7.1% (− 20.6 to 6.3%)	> 0.9999

^a^Matched prognostic factors: age, sex, vaccination status, immunocompromised status, and number of risk factors for progression to severe COVID-19

^b^P values computed from Fisher’s exact test

## Discussion

Though sotrovimab has clinical trial data demonstrating efficacy in reducing risk of hospitalization or death in at-risk COVID-19 outpatients [17], there were concerns of real-world effectiveness since the Omicron surge in late 2021, and its subvariants [1].

Our propensity-matched study showed no statistically significant differences in 30-day hospitalization, ICU admission and mortality rates between sotrovimab-treated patients with BA.2 versus BA.1 infection. All the estimates favoured better outcomes in the BA.2 group. As an example, in the propensity-matched group, the absolute risk for hospitalization in the BA.2 group was 14.3% less than the BA.1 group. However, there is uncertainty in this estimate as the 95% confidence interval is wide and ranges from 32.6% less to 4% more risk.

Of note, our findings are corroborated by two other studies. A French multicentre prospective cohort study (ANRS 0003S CoCoPrev) of high-risk mild-to-moderate COVID-19 patients showed low rates of disease-related hospitalization at day 28 following sotrovimab administration in 1/42 BA.2 (2.4%, 95% CI: 0–13%) and 3/125 BA.1-infected patients (2.4%, 95% CI: 1–7%), and no deaths [9]. However, the study results were not adjusted for baseline risks.

An English retrospective cohort study of COVID-19 outpatients treated with sotrovimab reported that 133 of 3,230 (4.1%) BA.1 cases and 140 of 3566 (3.9%) BA.2 cases were hospitalized with an adjusted hazard ratio of 1.02 (95% CI: 0.70–1.47) [8]. Although this study adjusted for age group and vaccination status to account for confounders, additional risk factors such as immunocompromised status were not accounted for.

When compared with other approved therapies for mild COVID-19, another study from the same French cohort (ANRS 0003S CoCoPrev) demonstrated similar COVID-19-related hospitalization rates 28 days following treatment administration between sotrovimab-treated (4/193) and nirmatrelvir/ritonavir-treated (0/55) cohorts (p = 0.24) [10]. The N gene Ct value slopes of both BA.1 and BA.2-infected patients were steeper amongst the nirmatrelvir/ritonavir-treated cohort, although the median time to negative PCR conversion amongst BA.2-infected patients did not significantly differ between the sotrovimab- and nirmatrelvir/ritonavir-treated groups [10]. This may be due to lack of statistical power from the relatively small number of nirmatrelvir/ritonavir-treated patients (in particular, BA.1-infected patients).

Our study’s strengths include its multicentre and propensity-matched study design where real-world comparative data on sotrovimab effectiveness against BA.2 versus BA.1 infections can be optimally estimated by risk differences with minimal baseline differences and biases.

### Limitations

Limitations included the small sample size—resulting in large confidence intervals for the calculated risk differences that cannot exclude small but clinically important differences—and the applicability of these findings for current Omicron subvariants. This was especially true for our BA.2 group (i.e., 15 patients), as our small sample size may fail to detect a signal of true differences between the two groups.

## Conclusion

In infectious diseases, in vitro assays have traditionally offered early insight into potential effectiveness of therapies such as monoclonal antibodies against emerging COVID-19 variants. The evidence for similar effectiveness of sotrovimab against BA.1 and BA.2 presents as an example where in vivo real-world clinical efficacy may potentially deviate from those initially suggested by in vitro data. More real-world data may be helpful to properly assess sotrovimab’s effectiveness against infections due to specific emerging COVID-19 variants, and whether they corroborate with laboratory data to translate to significant clinical differences for patient relevant outcomes.

### Supplementary Information

**Additional file 1.** Supplementary material including: **Text S1.** Criteria for immunocompromised conditions and risk factors for progression to severe COVID-19, based on local Canadian guidelines. **Table S1.** Balance of prognostic factors before and after matching by propensity scores. **Figures S1 and S2.** Graphical representation of unadjusted and adjusted risk differences of co-primary outcomes 30 days post-sotrovimab.

## Data Availability

The datasets used and/or analyzed during the current study are available from the corresponding author on reasonable request.

## Abbreviations

CI: Confidence interval
COVID-19: Coronavirus disease 2019
ICU: Intensive care unit
PCR: Polymerase chain reaction
SNP: Single-nucleotide polymorphism
WGS: Whole genome sequencing
