# Supplementary material for: Evaluating in vivo effectiveness of sotrovimab for the treatment of Omicron subvariant BA.2 versus BA.1: a multicentre, retrospective cohort study
Source: BMC Res Notes. 2024 Jan 24;17:37. doi: 10.1186/s13104-024-06695-x (PMC10809552; doi:10.1186/s13104-024-06695-x)
Supplement: Supplementary file 1 — Additional file 1. Supplementary material including: Text S1. Criteria for immunocompromised conditions and risk factors for progression to severe COVID-19, based on local Canadian guidelines. Table S1. Balance of prognostic factors before and after matching by propensity scores. Figures S1 and S2. Graphical representation of unadjusted and adjusted risk differences of co-primary outcomes 30 days post-sotrovimab. [file 13104_2024_6695_MOESM1_ESM.pdf]

Supplementary material

Lo et al. – Evaluating *in vivo* effectiveness of sotrovimab for the treatment of Omicron subvariant BA.2 versus BA.1: a multicentre, retrospective cohort study

Table of Contents

Text S1 Criteria for immunocompromised conditions and risk factors for progression to severe COVID-19, based on local Canadian guidelines [1].. 2

Table S1 Balance of prognostic factors before and after matching by propensity scores 3

Figure S1 Risk differences of co-primary outcomes 30 days post-sotrovimab (BA.1 vs. BA.2) 4

Figure S2 Adjusted risk differences of co-primary outcomes after matching by propensity score............. 5

Text S1 Criteria for immunocompromised conditions and risk factors for progression to severe COVID-19, based on local Canadian guidelines [1]

1) Immunocompromised condition
- Solid organ transplant
- Haematopoietic stem cell transplant
- Receipt of CAR-T therapy
- Haematologic malignancy
- Moderate or severe primary immunodeficiency
- HIV that is untreated or advanced AIDS
- High dose corticosteroids >=20mg of prednisone equivalent for >2 weeks
- Treatment of solid tumour cancer
- Chemotherapy agents: alkylating agents, anti-metabolites
- Biologic agents e.g., tumor necrosis factor (TNF) blockers
- DMARD agents for rheumatologic conditions

2) Risk factors for severe COVID-19
- Obesity BMI >=30kg/m^2
- Diabetes mellitus
- Hypertension
- Heart disease e.g., coronary artery disease
- Congestive heart failure
- Chronic respiratory disease e.g., chronic obstructive pulmonary disease, cystic fibrosis
- Cerebral palsy
- Intellectual disability
- Sickle cell disease
- Moderate to severe chronic kidney disease eGFR <60mL/min
- Moderate to severe liver disease e.g., Child Pugh Class B or C cirrhosis
- Currently pregnant

Reference:

1. Ontario COVID-19 Drugs and Biologics Clinical Practice Guidelines Working Group. Clinical practice guideline summary: recommended drugs and biologics in adult patients with COVID-19 (version 10.0). Ontario COVID-19 Science Advisory Table. 2022. https://doi.org/10.47326/ocsat.cpg.2022.10.0. Accessed Jun 7, 2023.

Table S1 Balance of prognostic factors before and after matching by propensity scores

	BA.1 (N=14)	BA.2 (N=14)	Standardized difference before matching	Standardized difference after matching
Age in years, mean (standard deviation, SD)	62.8 (22.1)	61.1 (16.8)	0.1358	0.0839
Female	4 (28.6%)	4 (28.6%)	0.5554	0
Received 2 or more doses of COVID-19 vaccines	12 (85.7%)	12 (85.7%)	0.1796	0
Immunocompromised	9 (64.3%)	9 (64.3%)	0.1488	0
Number of risk factors for progression to severe disease, mean (SD)	2.8 (1.7)	2.8 (1.7)	0.6211	0

Figure S1 Risk differences of co-primary outcomes 30 days post-sotrovimab (BA.1 vs. BA.2)

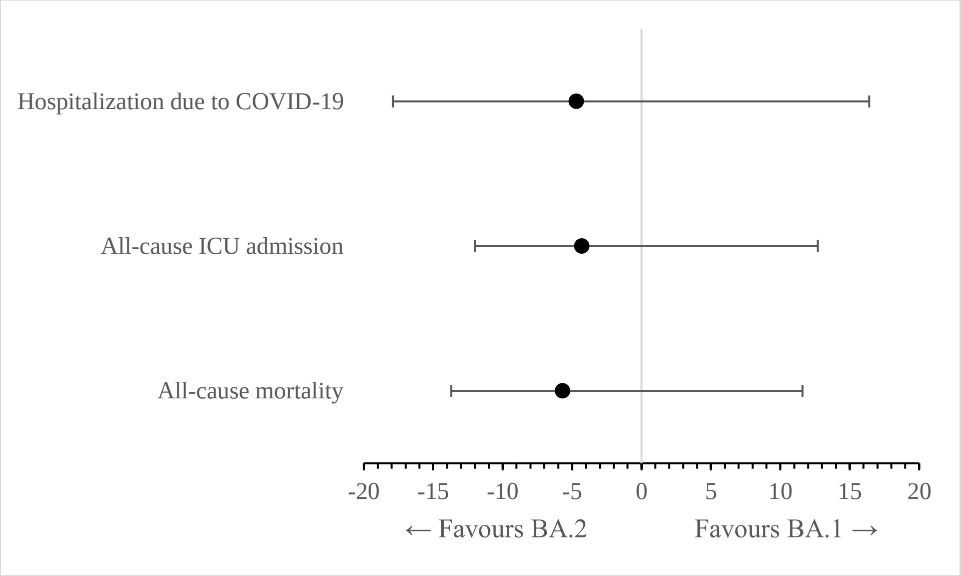

Graphical representation of unadjusted risk differences of co-primary outcomes (**Table 2**), presented as BA.2 minus BA.1 with two-sided 95% confidence intervals

Figure S2 Adjusted risk differences of co-primary outcomes after matching by propensity score

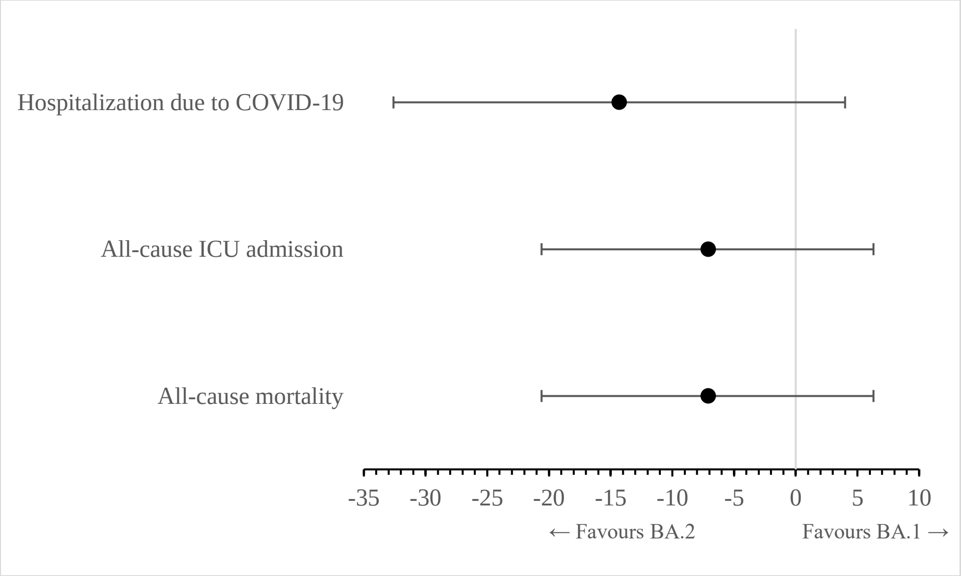

Graphical representation of adjusted risk differences of co-primary outcomes after matching by propensity score[a] (**Table 3**), presented as BA.2 minus BA.1 with two-sided 95% confidence intervals

[a]Matched prognostic factors: age, sex, vaccination status, immunocompromised status, and number of risk factors for progression to severe COVID-19
